# Computational modeling of the mechanical modulation of the growth plate by sustained loading

**DOI:** 10.1186/1742-4682-9-41

**Published:** 2012-09-25

**Authors:** Carlos A Narváez-Tovar, Diego A Garzón-Alvarado

**Affiliations:** 1 Mechanical engineering applications and research group (GEAMEC), Universidad Santo Tomás, Cra 9 No. 51-11, Bogotá, Colombia; 2 Research group on numerical methods for engineering (GNUM), Universidad Nacional de Colombia, Cra 30 No. 45-03, Bogotá, Colombia

**Keywords:** Growth plate, Chondrocytes, Endochondral ossification, Mechanical modulation, Computational model

## Abstract

This paper presents a computational model that describes the growth of the bone as a function of the proliferation and hypertrophy of chondrocytes in the growth plate. We have included the effects of the mechanical loads on the sizes of the proliferative and hypertrophic areas, the number of proliferative chondrocytes and the final size of the hypertrophic chondrocytes. The validation of the model was performed with experimental data published on other investigations about proximal tibia of rats, subjected to sustained axial stresses of 0.1 MPa, 0.0 MPa, -0.1 MPa and −0.2 MPa. Growth was simulated during 23 days, obtaining numerical errors between 2.77% and 3.73% with respect to experimental growth rates. The results obtained show that the model adequately simulates the behavior of the growth plate and the effect of mechanical loads over its cellular activity.

## Introduction

The longitudinal growth of long bones is due to the cellular activity of their two growth plates, one for each end. The growth plate is the cartilage essential structure for endochondral ossification, in which chondrocytes are found in reserve, proliferation and hypertrophy states. These cell states define each of the zones of the growth cartilage, whose width can vary between species [1].

The cellular activity of the growth plate is regulated by systemic and local factors, both biochemical and mechanical type. The most important local biochemical factor is the negative activator-inhibitor loop formed by the parathyroid hormone-related protein (PTHrP) and the Indian hedgehog (Ihh) [2]: While the PTHrP negatively regulates chondrocyte hypertrophy, Ihh positively regulates the entry of chondrocytes in the proliferative zone. Additionally there are other biochemical factors that influence endochondral ossification, such as BMP and FGFs, among others.

On the other hand, it is known that mechanical loads regulate the growth rate. Both the work of Delpech [3] and the Heuter-Volkmann law establish that compression retards growth, while distraction promotes it. Experiments [4-6] have shown that mechanical loads produces several alterations in the growth plate, including changes in the width of the hypertrophic and proliferative zones, the final size of the hypertrophic chondrocytes and the number of new cells produced. Among all of these alterations, the one that has greater influence on the growth rate is the change in the final size of the hypertrophic chondrocytes [6].

The behavior of the growth plate during endochondral ossification has been modeled using the finite element method. The models used can be classified according to the regulatory factor involved. The models of biochemical regulation, as the ones developed by Brouwers et al. [7] or Garzón-Alvarado et al. [8], take into account the PTHrP-Ihh regulatory loop and its effect on the differentiation of chondrocytes from proliferative to hypertrophic state. On the other hand, the most representative models of mechanical regulation have been developed by Stokes et al. [6] and Carter et al. [9]. The model of Carter et al. [9] describes an isotropic growth tensor as a function of the deviatoric and hydrostatic stress components. For its part, the model of Stokes et al. [6] is based on experimental evidence and describes the longitudinal bone growth depending on the tensile or compressive axial stresses. Both models were compared by Lin et al. [10] by simulating growth in a human T7 vertebra under different loading conditions. The comparison established that the model of Carter et al. [9] do not intrinsically involve the preferential direction of growth, while the model of Stokes et al. [6] is limited to loading conditions that do not involve shear stresses.

The main objective of this work is to develop, from the biological aspect, a finite element model that simulates the behavior of the growth plate subjected to sustained tensile or compressive loading. We programmed a two-dimensional plane strain element with four nodes, which elongates in the preferential growth direction depending on the cellular differentiation cycle and the final size of the hypertrophic chondrocyte. These are functions of the external mechanical loads applied to the growth plate. In order to validate the model we used the data found in Stokes et al. [5] for the proximal tibia of rats subjected to sustained stresses of −0.2 MPa, -0.1 MPa, 0.0 MPa and 0.1 MPa. We used a square domain of 800 μm of side, which includes the changes produced on the width of the proliferative and hypertrophic zones. Simulations of bone growth during 23 days were performed, obtaining accumulated numerical errors between 2.77% and 3.73%, compared to experimental growth rates [5,11]. The results obtained show that the model adequately simulates the behavior of the physis and its mechanical modulation by sustained axial stress.

## Materials and methods

### Mathematical model

The main hypothesis of the proposed model is: the bone elongation is mainly due to the proliferation and hypertrophy of chondrocytes that are organized by columns in the growth plates. Both cellular processes (proliferation and hypertrophy) are regulated by biochemical and mechanical factors [12]. We describe below the mathematical model used.

### Biochemical factors

The model considers that the main biochemical factor is the PTHrP-Ihh regulatory loop. Also, it is assumed that its expression mechanism is insensitive to mechanical loads [13]. The above is based on the temporal stability of the Turing pattern formed by the PTHrP-Ihh reaction–diffusion system and on the in vitro results reported by Villemure et al. [14].

### The growth rate

It is assumed that there is no extracellular matrix between hypertrophic chondrocytes in the preferential growth direction [6]. Moreover, in steady state, all the new chondrocytes complete their cell cycle from the proliferative to hypertrophic state. Therefore, the growth rate *G* can be expressed as:

(1) $$G=n_{p}\times h_{\max}$$

where *n*_*p*_ is the number of chondrocytes that proliferate per unit of time (cells/day) and *h*_*max*_ is the maximum size, in the direction of growth, that reach the chondrocyte in the hypertrophic zone.

### The chondrocyte distribution and concentration

According to the model proposed by Garzón-Alvarado et al. [12], the description of the cellular distribution and concentration over the growth plate can be performed through two transversely isotropic second-order tensors, defined as

(2) $$R_{\mathrm{PC}}=\sqrt[{3}]{\frac{C_{\mathrm{PC}}}{r_{\mathrm{PC}}}}\left(1+\left(r_{\mathrm{PC}}-1\right)n⊗n\right)$$

(3) $$R_{\mathrm{HC}}=\sqrt[{3}]{\frac{C_{\mathrm{HC}}}{r_{\mathrm{HC}}}}\left(1+\left(r_{\mathrm{HC}}-\right)n⊗n\right)$$

where **R**_*PC*_ is the distribution tensor for the chondrocytes in the proliferative zone, *r*_*PC*_ is the ratio of the number of proliferative chondrocytes in the preferential growth direction (**n**) with respect to the number of cells in the orthogonal direction and *C*_*PC*_ is the concentration of chondrocytes in the proliferative zone. Similarly, **R**_*HC*_ is the distribution tensor for the chondrocytes in the hypertrophic area, *r*_*HC*_ is the ratio of the number of hypertrophic chondrocytes in the preferential growth direction (**n**) with respect to the number of cells in the orthogonal direction and *C*_*HC*_ is the concentration of chondrocytes in the hypertrophic zone. Finally, **1** is the identity tensor of second order.

### The growth tensor

The growth rate tensor $\dot{\varepsilon }$ can be written as:

(4) $$\dot{\varepsilon }=d^{\mathrm{proliferation}}+d^{\mathrm{hypertrophy}}$$

where **d**^*proliferation*^ and **d**^*hypertrophy*^ are the strain rate tensors due to proliferation and hypertrophy of chondrocytes, respectively.

Growth in the proliferative zone is due to cellular mitosis, therefore **d**^*proliferation*^ is given by:

(5) $$d^{\mathrm{proliferation}}=\left(n_{p}\frac{h_{p}}{l_{p}}\right)n⊗n$$

where *h*_*p*_ is the size of the proliferative chondrocyte and *l*_*p*_ is the width of the proliferative zone.

Furthermore, the growth in the hypertrophic zone is due to elongation experienced by chondrocytes during its maturation from the proliferative to the hypertrophic state [12]:

(6) $$d^{\mathrm{hypertrophy}}=\left(\frac{1}{l_{h}}\underset{i\in C_{H}}{\sum }\left(\frac{h_{i}\left(t\right)-h_{p}}{\text{Δ}t_{i}}\right)\right)n⊗n$$

where *h*_*i*_ is the instantaneous size of the i^th^ chondrocyte once hypertrophy begins, *Δt*_*i*_ is the elapsed maturation time necessary to reach the height *h*_*i*_, and *l*_*h*_ is the width of the hypertrophic zone.

### The size of the hypertrophic chondrocyte

Taking into account that the maximum height of the hypertrophic chondrocyte depends on the mechanical loads [4], we propose a growth function of *h*_*i*_ that depends on the change of stress in the preferential direction of growth *σ*_*n*_

(7) $$h_{i}\left(t\right)=h_{p}+\frac{h_{\max}\left(\text{Δ}\sigma_{n}\right)-h_{p}}{t_{E}}\Delta t_{i}$$

where *t*_*E*_ is the maximum time required for a proliferative chondrocyte to mature into a fully hypertrophic one [12]. The maximum size of the hypertrophic chondrocyte *h*_*max*_ can be expressed as:

(8) $$h_{\max}=\left(1+D\_h_{\max}\left(\text{Δ}\sigma_{n}\right)\right)h_{\max}^{f}$$

where *h*_*max*_^*f*^ is the maximum size of the hypertrophic chondrocyte under physiological loading conditions, *D*_*h*_*max*_ is the change in the maximum size of hypertrophic chondrocyte due to sustained mechanical loading and it has to be obtained experimentally. Finally, *Δσ*_*n*_ is the difference between the actual stress in the preferential direction of growth *σ*_*n*_ and the stress under physiological conditions *σ*_*n*_^*f*^:

(9) $$\text{Δ}\sigma_{n}=\sigma_{n}-\sigma_{n}^{f}$$

Under physiological conditions, observe that *σ*_*n*_ = *σ*_*n*_^*f*^, thus *Δσ*_*n*_ = 0.

### Computational implementation

**Figure 1:**
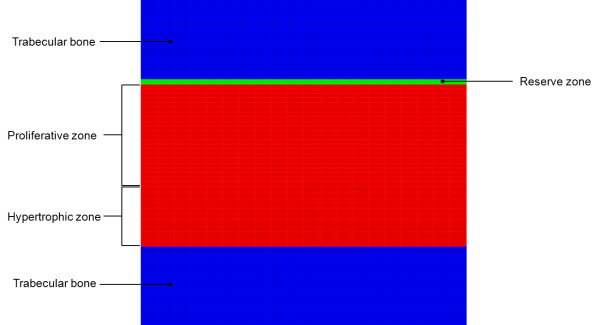
Two-dimensional domain used for the computational implementation.

**Figure 2:**
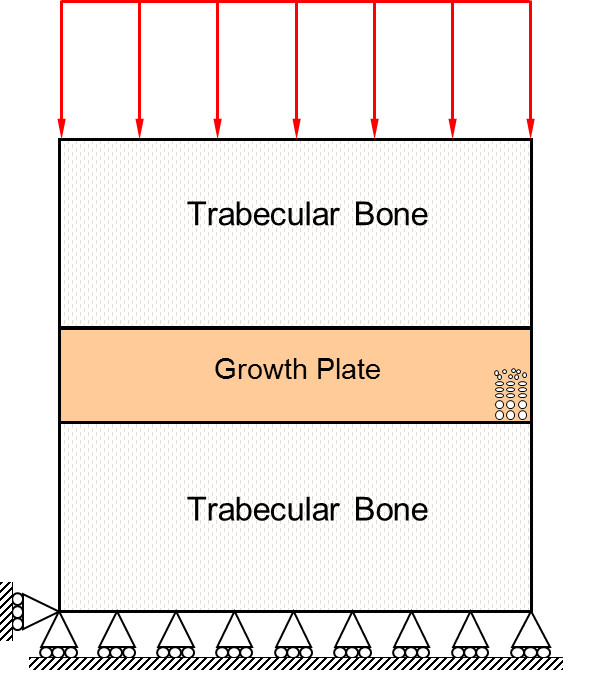
Support conditions and sustained load used in the simulation.

The equations described above were solved numerically using the finite element method. We implemented a two-dimensional plane strain element with four nodes. We used a square domain divided into five regions (Figure 1): the upper and lower zones correspond to the epiphyseal and metaphyseal trabecular bone, both with the same thickness, while the inner regions correspond to the hypertrophic, proliferative and reserve cartilage zones. Figure 2 illustrates the support conditions and sustained load used in the simulation.

It is known that the thicknesses of the growth plate and their columnar zones change due to mechanical loading [5], thus the domain was parameterized in order to update the geometry according to $\Delta \sigma_{n}$. On the other hand, the finite element mesh was parameterized according to the cellular concentration and distribution at each cartilage zone, in such a way that for each element in the proliferative zone *r*_*PC*_ = 1/1. Similarly, for each element of the hypertrophic zone *r*_*HC*_ = 1/1.

An incremental iterative scheme was used, in which the time interval is defined by the rate of production of new cells in the proliferative zone and by the maximum size of hypertrophic chondrocytes. Assuming that the growth plate is at equilibrium, the rate of cell production must equal the rate of chondrocytes undergoing apoptosis in the hypertrophic zone, therefore the time interval is defined by:

(10) $$\text{Δ}t=\frac{h_{\max}}{G}$$

Note that a new cell is produced in the proliferative zone and a mature chondrocyte suffers apoptosis in the hypertrophic zone in each iteration. Since the growth rate changes due to mechanical loading [5], a different time interval has to be used for each loading condition.

### Mechanical tissue properties

Following a similar approach to the one proposed by Piszczatowski [15], all tissues were treated as linear isotropic materials and their mechanical properties are summarized in Table 1. We included the change of mechanical properties within the growth plate, according to the experimental study of Sergerie et al. [16]. 

**Table 1:** Mechanical properties for each of the tissues

**Type of Tissue**	**E [MPa]**	**ν**	**Reference**
Trabecular bone	2000	0.30	Sylvestre et al. [17]
Reserve zone cartilage	0.48	0.07	Sergerie et al. [16]
Columnar cartilage – proliferative zone	0.25	0.13	Sergerie et al. [16]
Columnar cartilage – hypertrophic zone	0.27	0.13	Sergerie et al. [16]

### Model validation

In order to validate the model, we simulated the growth of the rat proximal tibia during 23 days. We decided to use these particular species and anatomical location because all parameters related could be obtained from published literature [5,11]. The cellular parameters under physiological conditions were obtained from the histology reported by Taylor et al. [11]. On the other hand, the parameters associated with the mechanically modulated growth under stress differences *Δσ*_*n*_ of −0.2 MPa, -0.1 MPa, 0.0 MPa and 0.1 MPa were calculated using the data reported by Stokes et al. [5].

### Thickness of the growth cartilage zones

Based on the thickness of each of the cartilage zones under physiological conditions, we calculate the thickness for each load condition using the percentage changes for sustained stresses of −0.2 MPa, -0.1 MPa, 0.0 MPa and 0.1 MPa [5]. The values are summarized in Table 2. 

**Table 2:** **Thicknesses of the cartilage zones for each load case, according to Taylor et al.**[11]**and Stokes et al.**[5]

	**Thickness of the cartilage zones [μm]**		
***Δσ***_***n***_**[MPa]**	**Reserve**	**Proliferative**	**Hypertrophic**
0.1	13.9	288	175
0.0	12.3	264	162
−0.1	11.9	269	129
−0.2	10.9	242	123

### Cellular distribution and concentration

We modeled 20 chondrocyte columns with a separation of 40 μm. At physiological conditions [11], each column has 22 proliferative chondrocytes and 6 hypertrophic chondrocytes with a maximum size *h*_*max*_^*f*^ of 35 μm. The number of cells per column for each loading condition, calculated with the experimental percentage changes [5], is summarized in Table 3. 

**Table 3:** **Cell distribution according to Taylor et al.**[11]**and Stokes et al.**[5]

***Δσ***_***n***_**[MPa]**	**Chondrocytes per Column**	
	**Proliferative**	**Hypertrophic**
0.1	23	7
0.0	22	6
−0.1	22	5
−0.2	18	5

### Size change of the hypertrophic chondrocyte

*D*_*h*_*max*_ is obtained by linear interpolation from the percentage change on the maximum size of the hypertrophic chondrocyte in the rat proximal tibia [5] for sustained stresses of −0.2 MPa, -0.1 MPa, 0.0 MPa and 0.1 MPa, as

(11) $$D\_h_{\max}=\{\begin{array}{ccc} 0.35\text{Δ}\sigma_{n} & \text{if} & \text{Δ}\sigma_{n}\geq 0\;\text{MPa}\\ -0.27\text{Δ}\sigma_{n} & \text{if} & -0.1\;\text{MPa}\geq \text{Δ}\sigma_{n}<0\;\text{MPa}\\ 0.47\text{Δ}\sigma_{n}+0.074 & \text{if} & \text{Δ}\sigma_{n}<-0.1\;\text{MPa} \end{array}$$

where *Δσ*_*n*_ is the magnitude of *Δσ*_*n*_.

### Time intervals

The growth rates and the maximum chondrocyte height were calculated using the percentage changes reported by Stokes et al. [5]. The resultant time intervals are reported in Table 4. 

**Table 4:** Time intervals used for the simulation

***Δσ***_***n***_**[MPa]**	***G***_***Stokes***_**[μm/day]**	***h***_***max***_**[μm]**	*Δ****t*[days]**
0.1	228.7	36.22	0.158
0.0	217.0	35.00	0.161
−0.1	183.4	35.94	0.196
−0.2	163.8	34.30	0.209

## Results

**Figure 3:**
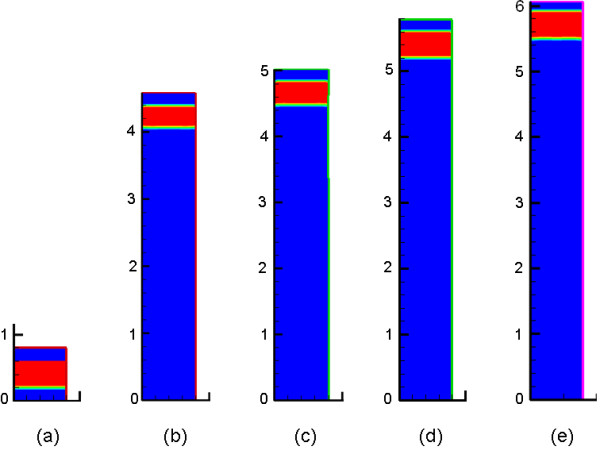
**Elongation of the domain under uniform axial load for 23 days (a) initial size and final sizes for (b)*Δσ***_***n***_**= − 0.2 MPa, (c)*Δσ***_***n***_**= − 0.1 MPa, (d)*Δσ***_***n***_**=0 MPa, and (e)*Δσ***_***n***_**=0.1 MPa.**

**Figure 4:**
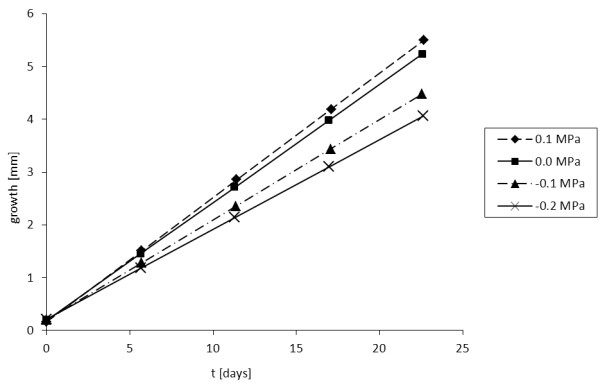
Growth of bone tissue under uniform axial distributed load during 23 days.

Figure 3 compares the elongation of the domain under stress changes *Δσ*_*n*_ of −0.2 MPa, -0.1 MPa, 0 MPa and 0.1 MPa for 23 days, while Figure 4 illustrates the graphs of bone growth during the same period of time. It is observed that the model is able to simulate the effect of the mechanical load on the growth plate and hence the modulation of the growth rate.

Table 5 compares the growth rates obtained with respect to those calculated using the percentage changes reported by Stokes et al. [5]. We can note that a numerical error was obtained between 2.77% for *Δσ*_*n*_ =0.1 MPa, and 3.73% for *Δσ*_*n*_ = − 0.2 MPa. 

**Table 5:** **Comparison of the growth rates obtained with respect to those calculated using the percentage changes by Stokes et al.**[5]

***Δσ***_***n***_**[MPa]**	***G*[μm/day]**	***G***_***Stokes***_**[μm/day]**	**Error [%]**
0.1	235.1	228.7	2.77
0.0	223.2	217.0	2.86
−0.1	189.6	183.4	3.42
−0.2	169.9	163.8	3.73

## Discussion

This paper presents a new computational model that describes the growth plate activity and simulates mechanically modulated bone growth under sustained axial loading. The inclusion of the growth plate alterations produced by mechanical loading is essential for the understanding of bone growth because we can describe and simulate the change of the growth rate, from a biological aspect, as a function of the chondrocyte proliferation and hypertrophy processes. In order to achieve that purpose, the model includes the growth plate alterations produced by axial sustained loading: the change in the thickness of the proliferative and hypertrophic cartilage was implemented by a parameterized two-dimensional domain; the change in the number of proliferative chondrocytes was implemented with a parameterized FE-mesh and the change in the final size of the hypertrophic chondrocyte was included as a growth function that depends on the stress in the preferential direction of growth. All of these alterations were described experimentally and reported in the published literature [4-6].

Since the description of the growth rate was made from the biological aspect, the validation of the model requires an extensively study of the growth plate alterations due to mechanical loading. We decided to validate the model by simulating the growth of the rat proximal tibia [5] during 23 days, under sustained stresses of −0.2 MPa, -0.1 MPa, 0 MPa and 0.1 MPa. We obtained growth rates with numerical errors between 2.77% and 3.73% respect to those that were calculated using the experimental data set. As expected [3-6], compression stresses over the physiological load condition retard bone growth while tensile stresses promote it. This is explained mainly by the changes in the size of the hypertrophic chondrocyte [5].

Although the computational model represents an advance in the computational mechanobiology of the growth plate, it has certain limitations that have to be discussed. As previously mentioned, the model requires experimental studies for quantifying the alterations of the growth plate due to mechanical loading. Those studies are impossible to be done on human beings, thus we will continue to depend on animal models. On the other hand, there are several studies published with data sets for other species and anatomical locations, but some of the quantified parameters are not the same as those required for the model, or the data sets are less complete than the rat proximal tibia. In addition, none of the studies report the mechanical properties of the analyzed growth plates, therefore the mechanical properties of the tissues were extracted from other papers [16,17] and treated as linear isotropic materials.

Other limitations are associated with the mathematical formulation: First, the model assumes that growth depends only on the proliferation and hypertrophy of chondrocytes, but ignores the synthesis and degradation of the extracellular matrix as another important factor for endochondral growth [1]. Second, the model includes the main growth plate alterations produced by the sustained loading, but it does not include the effects of dynamic loading. Finally, the model was implemented in a two-dimensional domain in which other important structures were not included, such as the ring of Lacroix. Despite these limitations, the model adequately simulates the cellular activity of the growth plate and its mechanical regulation by sustained loading, therefore the numerical errors obtained can be considered acceptable.

Future research will be focused on extending the model to the computational analysis of the mechanical treatments prescribed to correct the developmental dysplasia of the hip. Tridimensional models of child dysplastic hips will be obtained by tomographic image reconstruction and then they will be loaded according to the different braces and harnesses that are commonly used. A multiscale model will be implemented in order to obtain the mechanical stimulus over the growth plate (at the macro level) and the evolution of the cellular processes (at the micro level).

## Competing interests

The authors declare that they have no competing interests.

## Authors’ contributions

All authors read and approved the final manuscript.
